# Assessing demographic and clinical determinants of resilience, personal recovery, and quality of life for psychiatric in-patients before hospital discharge

**DOI:** 10.1192/j.eurpsy.2025.479

**Published:** 2025-08-26

**Authors:** E. Owusu, W. Mao, R. Shalaby, H. Elgendy, B. Agyapong, E. Eboreime, M. A. Lawal, N. Nkire, C. T. Hilario, P. Silverstone, P. Chue, X.-M. Lin, Y. Wei, W. Vuong, A. Ohinmaa, V. Taylor, A. J. Greenshaw, V. I. Agyapong

**Affiliations:** 1 Psychiatry, University of Alberta, Edmonton; 2 Psychiatry, Dalhousie University, Halifax; 3 Nursing, University of British Columbia, Okanagan; 4 Addiction and Mental Health, Alberta Health Services; 5 School of Public Health, University of Alberta, Edmonton; 6 Psychiatry, University of Calgary, Calgary, Canada

## Abstract

**Introduction:**

Many patients with mental health and emotional problems often see the transition period in the community after hospital discharge as a test of their resilience and a threat to their recovery. Most often, some doubt their ability to cope with the everyday challenges that may confront them in the community.

**Objectives:**

This paper assesses how demographic and clinical characteristics predicted resilience, personal recovery and quality of life.

**Methods:**

Data were collected from psychiatric inpatients prior to their discharge into the community using the REDCap, an online survey platform. Resilience, personal recovery, and quality of life were assessed using the Brief Resilience Scale (BRS), Recovery Assessment Scale (RAS), and EQ-Visual Analogue Scale (EQ-VAS), respectively. One-way analysis of covariance between groups (ANCOVA) was conducted to compare the relationships between groups. The dependent variables comprised mean scores of BRS, RAS and EQ-VAS. Demographic and clinical variables such as age, gender, ethnicity, and mental health diagnosis groups were independent variables, and covariates comprised demographic/clinical factors such as gender, ethnicity, and mental health diagnosis

**Results:**

The survey results indicate that males had significantly higher resilience scores compared to females ( Mdiff = 0.270, CI= 0.144– 0.397, p=.<.001) and others (Mdiff =0.470, 0.093- 0.846, p=<.001); Black people indicated significantly higher quality of life than Caucasians (Mdiff = 8.79, 2.73- 14.85, P= <.001), and Indigenous people (Mdiff = 14.50, 6.45 - 22.51, p=<.001), respectively. In terms of relative recovery, participants with depression had significantly lower recovery compared to those with bipolar disorder (Mdiff = -10.25, -14.40- -6.10, p=<.001), schizophrenia (Mdiff f = -8.60, -13.20- -3.99, p=<.001), and substance use disorder (Mdiff = -8.30, -15.50- -1.42, p=<.005).

**Conclusions:**

The present results indicate that women, younger adults, and Indigenous peoples may be more challenged in adapting to the challenges of post-discharge life in the community. Our data may be helpful in communicating to policymakers and providers of funds the need to implement and evaluate outcomes of inpatient and community programs focusing on supporting resilience to improve recovery outcomes after discharge from the patient setting.

**Disclosure of Interest:**

None Declared

